# Ganglioside GM3 Levels Are Altered in a Mouse Model of HIBM: GM3 as a Cellular Marker of the Disease

**DOI:** 10.1371/journal.pone.0010055

**Published:** 2010-04-07

**Authors:** Thomas Paccalet, Zoé Coulombe, Jacques P. Tremblay

**Affiliations:** Centre de Recherche du Centre Hospitalier Universitaire de Québec - Unité de Génétique Humaine, Université Laval, Québec, Canada; McMaster University, Canada

## Abstract

**Objective:**

HIBM (Hereditary Inclusion Body Myopathy) is a recessive hereditary disease characterized by adult-onset, slowly progressive muscle weakness sparing the quadriceps. It is caused by a single missense mutation of each allele of the UDP-N-acetylglucosamine 2-epimerase/N-acetylmannosamine kinase (GNE) gene, a bifunctional enzyme catalyzing the first two steps of sialic acid synthesis in mammals. However, the mechanisms and cellular pathways affected by the GNE mutation and causing the muscle weakness could not be identified so far. Based on recent evidence in literature, we investigated a new hypothesis, i.e. the involvement in the disease of the GM3 ganglioside, a specific glycolipid implicated in muscle cell proliferation and differentiation.

**Methods:**

qRT-PCR analysis of *St3gal5* (GM3 synthase) gene expression and HPLC quantification of GM3 ganglioside were conducted on muscle tissue from a mouse model of HIBM harboring the M712T mutation of GNE (*Gne^M712T/M712T^* mouse) vs control mice (*Gne^+/+^* mouse).

**Results:**

*St3gal5* mRNA levels were significantly lower in *Gne^M712T/M712T^* mouse muscles vs *Gne^+/+^* mouse muscles (64.41%±10% of *Gne^+/+^* levels). GM3 ganglioside levels showed also a significant decrease in *Gne^M712T/M712T^* mouse muscle compared to *Gne^+/+^* mouse muscle (18.09%±5.33% of *Gne^+/+^* levels). Although these *Gne^M712T/M712T^* mice were described to suffer severe glomerular proteinuria, no GM3 alterations were noted in kidneys, highlighting a tissue specific alteration of gangliosides.

**Conclusion:**

The M712T mutation of GNE hampers the muscle ability to synthesize normal levels of GM3. This is the first time that a mutation of GNE can be related to the molecular pathological mechanism of HIBM.

## Introduction

Hereditary Inclusion Body Myopathy (HIBM, OMIM600737, inclusion body myopathy 2, Distal Myopathy with Rimmed Vacuoles) is a unique autosomal recessive muscle disorder, characterized by adult-onset of muscle weakness in upper and lower limbs. Amazingly, the quadriceps seemed to be spared until late stages of the disease [1], [2], although recent evidence suggests that rectus femoris could be affected [3]. HIBM has been originally described in Japanese [4] and Iranian Jews [1] but patients are now found worldwide. HIBM muscle fibres present typical pathology, with rimmed vacuoles and cytoplasmic and/or nuclear filamentous inclusions. Intracellular deposits of β-amyloid proteins, altered phosphorylation of tau or activation of the ubiquitin proteasome or of the lysosomal systems can occasionally be observed [1], [5]–[7].

HIBM was mapped to chromosome 9 [8], [9] and is associated with mutations in the UDP-N-acetylglucosamine 2-epimerase/N-acetylmannosamine kinase (*Gne*) gene [8]. This gene encodes a bifunctional enzyme (hereafter designed as GNE) catalyzing the epimerisation of UDP-N-acetylglucosamine (UDP-GlcNAc) in N-acetylmannosamine (ManNAc) and then the phosphorylation of ManNAc in ManNAc-6-phosphate (ManNAc-6P). These are the first two steps of N-acetylneuraminic acid (Neu5Ac) biosynthesis [10], the most abundant terminal sialic acid of the eukaryote glycoconjugates. Neu5Ac is implicated in numerous cellular functions, such as cell-cell interactions, signalling, haematopoietic cell differentiation as well as regulation of the immune system [11]–[13]. Knock-out of *Gne* gene causes embryonic lethality in mice at day 8.5, highlighting the importance of sialylation [14].

GNE activities rely in two functional domains, controlling respectively the epimerase and the kinase activities. Over 40 different mutations spreading over both domains of *Gne* can cause HIBM. For example, the most prevalent mutations of *Gne* gene, M712T and V572L respectively in the Middle Eastern community and in Japanese patients, are both located in the kinase domain of GNE, but other mutations have been identified in its epimerase domain. All these mutations of *Gne* gene result in different altered enzymatic activities [15]–[18], which should lead to lessened status of cellular sialylation or at least reduced sialylation of specific glycoproteins, such as α-dystroglycan [19], [20], NCAM [21], neprilysin [22] or aberrant activity of global systems like lysosomal activity and trafficking [7], [23], [24]. However, the impact of HIBM mutations on the overall sialylation in humans remains unclear [15], [16], [25]. So far, none of these works could identify the molecular pathological mechanism of HIBM and furthermore, a recent model of heterozygous GNE-deficient mice provided clues that cells and organs can manage a certain amount of defects in sialylation, at least down to an overall 25% reduction [26]. Moreover, sialic acids can be provided by exogenous sources and it is known that cells, be it mammalian or insect cell lines, can use circulating sialic acids to compensate their lack of this monosaccharide [16], [27], [28]. The context in which experiments are conducted is thus an important factor in the interpretation of the results. Other studies aimed at discovering cellular processes altered in this myopathy [29]–[31] have not proven anymore successful and the molecular aspects of the disease remain obscure yet.

Until now, the process by which mutations of GNE causes the muscular pathology observed in HIBM remains misunderstood. As all the characterized mutations affect differently the enzymatic activity of GNE, but result in similar phenotypes, it is questionable that an altered enzymatic function of GNE triggers the disease mechanism. It is thus possible that HIBM may be a consequence of the alteration of another yet unknown role of GNE.

Interestingly, GNE has a dynamic cellular localization, as it can be detected in the cytosol and the nucleus simultaneously [32]. The nuclear localization, which is not required for ManNAc-6P production, suggests alternative roles of GNE, other than the sole pathway of sialic acid biosynthesis. Recently, several articles underlined this hypothesis. GNE was proven to be involved in protein-protein interactions with collapsin response mediator protein 1 (CRMP1) and promyelocytic leukemia zinc finger protein (PLZF)[33], the later being a transcription factor whose knock-out alters musculoskeletal development in mice [34], and with alpha-actinin 1 [35], which helps to anchor the myofibrillar actin filaments.

GNE was also reported to regulate ganglioside (sialylated glycosphingolipids) levels in HEK293T cells, especially GM3 and GD3 [36]. In particular, cellular levels of GNE were shown to regulate beta-galactoside alpha-2,3-sialyltransferase 5 expression (ST3Gal5, the GM3 synthesizing enzyme). GM3 is the most abundant ganglioside in muscle, representing almost 70% of total ganglioside in muscle membranes [37]–[40]. Gangliosides, and particularly GM3 and GD3 are involved in regulation of numerous cellular pathways, such as migration, proliferation, senescence and apoptosis [41]–[43] and ganglioside levels change over time in muscle cells, particularly during differentiation [44]–[46]. Thus, an alteration of the capacity of GNE to effectively regulate GM3 synthesis could alter progressively the ability of muscle cells to adapt to their environment or to cope with their needs. To date, no investigation has been made to determine a relation between the ganglioside synthesis regulated by GNE and HIBM.

In 2007, two different genetically engineered mouse lines of mutations in the GNE gene were described, harbouring either M712T [47] or D176V [48] mutation. Both of these models aimed at mimicking HIBM phenotype in animals, yet M712T mice mutation lead to glomerular proteinuria, a pathology not described in human HIBM patients. This surprising lethal renal phenotype hindered the authors in their assessment of myopathic features in these mice. However, the M712T strain of mice could still help to understand specific alterations in muscles. In particular, we investigated whether mutations in GNE could alter the GM3 ganglioside levels of muscles and, consequently, the insulin response of muscular cells of M712T mice.

## Results

### Down-regulation of ST3Gal5 expression in *Gne^M712T/M712T^* mouse muscle

**Figure 1:**
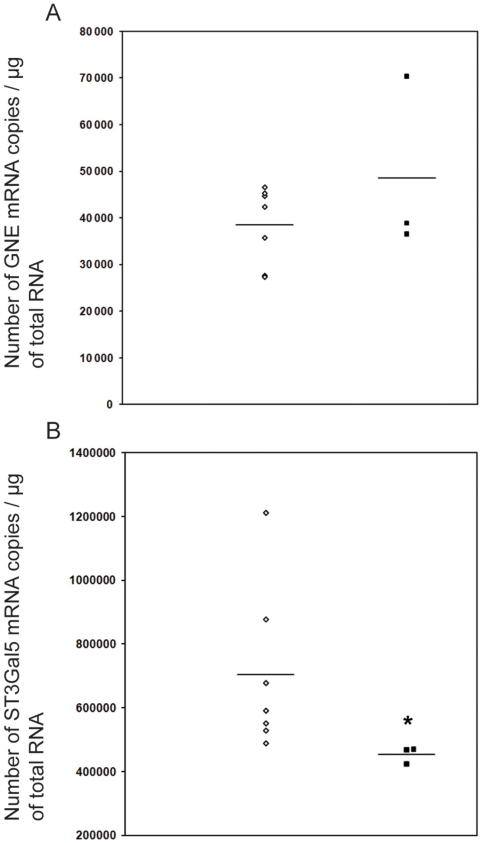
Effects of mutated GNE^M712T^ on ST3Gal5 mRNA expression. *A*, Comparison of GNE mRNA levels between *Gne^+/+^ (diamond)* and *Gne^M712T/M712T^ (square)* mouse muscle determined by qRT-PCR (absolute values are expressed as the number of GNE mRNA copies/µg of total RNA). *B*, ST3Gal5 mRNA levels *Gne^+/+^* and *Gne^M712T/M712T^* mouse muscle (absolute values are expressed as the number of GNE mRNA copies/µg of total RNA). (*, *p<0.025*); each data point represents a minimum of 3 replicates). Mean ST3Gal5 expression in *Gne^M712T/M712T^* muscle was 64.41%±10% of *Gne^+/+^* muscle, whereas no significant difference could be detected in GNE expression.

**Figure 2:**
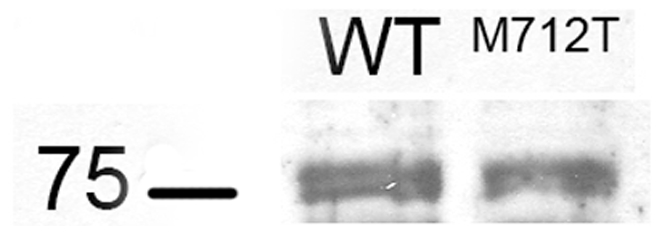
Immunoblotting of muscle of *Gne^+/+^* and *Gne^M712T/M712T^* mice. Immunoblots of muscle extracts from *Gne^+/+^* and *Gne^M712T/M712T^* showed no apparent difference in GNE protein expression (upper band, arrow, 79 kDa) in *Gne^M712T/M712T^* mice compared with *Gne^+/+^* littermates.

A recent study [36] pointed out that *Gne* expression levels were directly able to regulate ST3Gal5 expression levels. ST3Gal5 is the enzyme responsible for ganglioside GM3 synthesis. So far, the possible relationship between GM3 synthesis and HIBM was never investigated, yet GM3 is a glycosphingolipid largely involved in differentiation of muscle cells [44]–[46] and, more generally, in numerous cellular pathways, such as migration, proliferation, apoptosis or insulin sensitivity [42], [43]. Therefore, an alteration of its synthesis could directly lead to a vast modification of the cell metabolism. Thus, it was of interest to test whether a single-nucleotide mutation of *Gne* gene could modify ST3Gal5 expression pattern. It was first necessary to compare the raw levels of GNE mRNA expression between mutated (*Gne^M712T/M712T^*) and wild-type (*Gne^+/+^*) mouse muscles. As shown in Figure 1a, no difference in GNE mRNA levels could be detected in mice harboring the mutation in regards to *Gne^+/+^* mice. This result is consistent with the *Gne^M712T/M712T^* knock-in construction, where the mutated *Gne* gene remains under its natural promoter. GNE protein levels in *Gne^M712T/M712T^* and *Gne^+/+^* muscle tissue were assessed by electrophoresis and provided us a complementary verification of comparable GNE levels between *Gne^M712T/M712T^* and *Gne^+/+^* mouse muscles (Figure 2).

An evaluation of ST3Gal5 mRNA levels was then performed in *Gne^M712T/M712T^* and *Gne^+/+^* muscle tissues by real-time RT PCR. As shown in Figure 1b, *St3gal5* gene expression decreased of 35.6%±10% in *Gne^M712T/M712T^* mice with respect to *Gne^+/+^* (p<0.025). This result underlines the role of GNE in the regulation of ST3Gal5 expression.

### GM3 ganglioside levels are affected in *Gne^M712T/M712T^* muscles, compared with *Gne^+/+^* muscles

**Figure 3:**
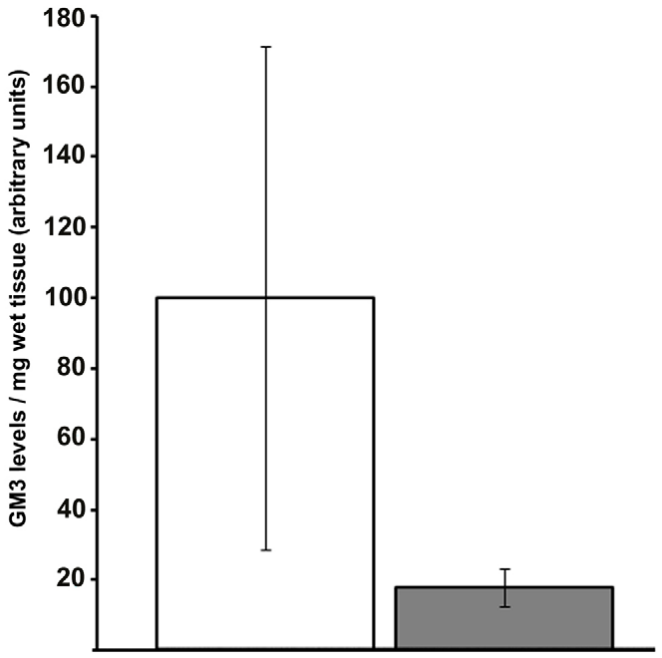
GNE^M712T^ muscles display changes to GM3 levels. *Gne^M712T/M712T^ (grey)* mouse muscle experienced reduced levels of GM3 gangliosides compared with *Gne^+/+^ (white)* muscle. HPLC data from a minimum of five replicates are pooled for the *Gne^+/+^* and *Gne^M712T/M712T^* samples (*, *p = 0.008751, Student t test*).

Next, we measured GM3 by HPLC to ensure that changes in ST3Gal5 mRNA levels were reflected in the actual cellular levels of the ganglioside produced by this enzyme. As described in Material & Methods, gangliosides were extracted by several solvent partitions and their oligosaccharides were digested by an endoglycoceramidase. The oligosaccharides were then derivatized with anthranilic acid and analyzed by HPLC, as previously demonstrated [49]. GD1b was used as internal standard, because endogenous GD1b was undetectable in muscle tissue samples (data not shown). Hereafter, ganglioside levels are reported as the ratio of the peak area of GM3 to that of internal standard GD1b (Figure 3). The GM3 levels were significantly lower in *Gne^M712T/M712T^* muscle than in *Gne^+/+^* muscle (18.09%±5.33% of *Gne^+/+^* levels, Student t test p = 0.008751).

### GM3 ganglioside levels seem unaffected in *Gne^M712T/M712T^* kidney, compared with *Gne^+/+^*

Ganglioside GM3 is particularly abundant in the kidney tissues [50]. Sialic acids are indeed important in kidneys, since their physicochemical properties are involved in the maintenance of the filtration barrier of glomeruli [51]. More particularly, changes in ganglioside expression in these organs is implicated in the pathogenesis of proteinuria [52]. More recently, it was demonstrated that alteration of ganglioside GM3 levels in kidneys can be related to glomerular hypertrophy and proteinuria [53].

**Figure 4:**
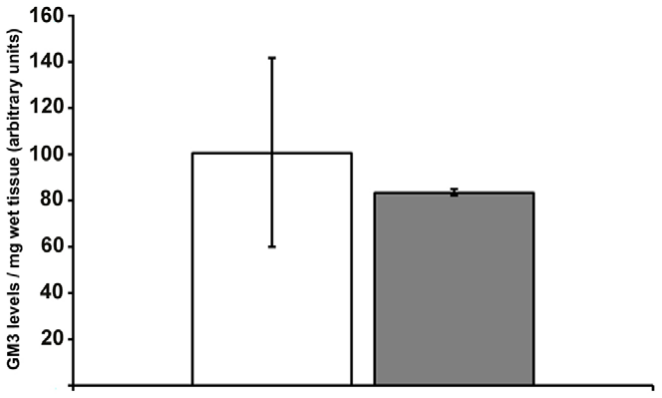
GNE^M712T^ kidney GM3 levels remain in normal range. *Gne^M712T/M712T^ (grey)* and *Gne^+/+^ (white)* mouse kidney gangliosides were analyzed by HPLC. No significant difference could be noted. HPLC data from a minimum of 3 replicates is pooled for the *Gne^+/+^* and *Gne^M712T/M712T^* samples.

However, our results clearly demonstrate that GM3 levels in *Gne^M712T/M712T^* mouse kidney are comparable with *Gne^+/+^* kidney levels (figure 4). Thus, GM3 should not represent an issue in the glomerular nephropathy reported in these mice by Galeano et al. [47].

## Discussion

One of the main problems when one tries to unravel the molecular and cellular pathways of HIBM is the lack of human muscle samples. Necessity arose to develop animal models of the mutations in the *Gne* gene. Two such models were developed to date and this study focuses on one of them, the *Gne^M712T/M712T^* mice.

*Gne^M712T/M712T^* mouse mutation leads to glomerular proteinuria [47], a pathology not described in human HIBM patients. This surprising lethal renal phenotype hindered the authors in their assessment of myopathic features in these mice. However, this strain of mice could still help us to understand specific alterations in muscle cell metabolism linked to GNE mutations. Galeano et al. [47] previously reported that the majority of these mice did not survive P3. In our colony, in contrast, several homozygous *Gne^M712T/M712T^* mice lived up to 6 months old. However the *Gne^M712T/M712T^* mouse phenotype remained altered in comparison with their littermates: *Gne^M712T/M712T^* mice were smaller than their control littermates, had a high mortality rate before weaning and presented renal abnormalities (data not shown). Moreover, in our colony, no difference could be observed between GNE protein levels of expression, here again in contrast with Galeano et al.

Our results demonstrate that in these mice, glycolipid sialylation, in particular ganglioside GM3 synthesis, is also affected. First, the *Gne^M712T/M712T^* mutation leads to a partial down-regulation of ST3Gal5 mRNA synthesis. However, according to Wang et al[36], when St3Gal5 mRNA levels were decreased to 12–15% of normal mRNA levels, the GM3 ganglioside levels remained 75% of normal levels. Thus, the sole effects of mutated GNE^M712T^ on the mRNA levels of ST3Gal5 (*i.e.* a decrease of 35.6%±10%) may not translate into such severe changes of GM3 in muscles (roughly 82% of diminution). However, it was reported that in *Gne^M712T/M712T^* mice, GNE^M712T^ enzyme activity showed only 19.4%±7.5% of GNE^WT^ activity [47]. Hence, the reduction of GM3 may result from a synergy of a lack of sialic acid and a blunt down-regulation of ST3Gal5.

Although beyond the scope of this article, these results emphasize the hypothesis formulated by Wang et al. [36] that GNE plays a role in transcription, most probably through its association with transcription factors, as described [33]. Future experiments with *Gne^D176V^* mice, a mouse strain harboring another mutation of GNE [48], could help us to determine whether different mutations of GNE all lead to an alteration of ST3Gal5 expression.

The rough lack in GM3 ganglioside in *Gne^M712T/M712T^* mice could have very well explained part of the renal pathologies observed in these animals, according to the relative abundance of GM3 in the kidney tissues [50]. Moreover, it was previously demonstrated that alteration of ganglioside GM3 levels in kidneys can lead to glomerular hypertrophy and proteinuria [53]. However, we could not detect any significant difference between GM3 levels in kidneys of GNE^M712T^ and GNE^WT^ mice. Such results suggest that GM3 alteration related to the GNE^M712T^ mutation could be specific to muscles, and would not affect other organs. This is of primary importance as HIBM patients usually do not develop pathologies in other tissues than muscles.

The present work emphasizes the fact that GNE is a moonlighting enzyme, capable of multiple intracellular roles, roles that only recent studies were able to discover [33], [35], [36]. The relationship between mutations of GNE and cellular levels of gangliosides was never explored before. Our results demonstrate that, beyond the production of sialic acid, the synthesis of specific molecules such as gangliosides, could be affected. Among gangliosides, GM3 is of particular interest, due to its preponderance in muscles [39]. Thus, an alteration of GM3 levels in HIBM cells could lead to a variety of detrimental effects and modification of cell metabolism and draw a bridge between several past observations on HIBM cells such as impairment of myogenic terminal differentiation (Amsili et al., personal communication) or modification of the apoptotic signaling pathways [54] as GM3 is precisely involved in terminal differentiation of myoblasts [44] and the control of apoptosis [36], [55].

In the case of muscle, lack of GM3 could not only modify the differentiation of cells but also their insulin related metabolism. Indeed, GM3 expression is directly related to insulin sensitivity [56], [57]. For instance, Type I Gaucher disease, which is due to impaired breakdown of GM3 and its accumulation in macrophages, is also associated with peripheral insulin resistance [58]. An enhanced insulin sensitivity was also reported in mice lacking GM3 [59]. Such modifications of GM3 expression and insulin sensitivity will soon be investigated thoroughly in the other available animal model of GNE mutation, developed by a Japanese team [48], [60] and in human cultured myoblasts.

If these different models all confirm that this ganglioside is altered when GNE is mutated, GM3 levels could then be tagged as a cellular marker of HIBM. Perhaps even more valuable, GM3 levels could be followed after genetic correction of myoblasts with healthy *Gne* gene and permit us to assess the phenotype reparation of cell. This would be the very first preliminary steps toward a cell therapy of HIBM.

## Materials and Methods

### Animals

The mouse model was produced by Dr Darvish (HIBM Research Group, Encino, USA), referred as *Gne^M712T/M712T^* mouse, with the most frequent mutation in the GNE gene in Jewish HIBM patients (M712T) is available in our laboratory. *Gne^M712T/M712T^* and wild-type *Gne^+/+^* mice were in C57BL/6 background strain. Animals were used in accordance with the animal protection committee of the CRCHUL. For all the experiments, *Gne^M712T/M712T^* and *Gne^+/+^* mice were paired for age (4 to 6 month old, mean age 5 month old).

### Primers

Primers were designed for mouse *Gne* and *St3gal5* (beta-galactoside alpha-2,3-sialyltransferase 5, SIAT9 or GM3 synthase) genes following Wang et al. [36] and adapted for murine genes. Oligonucleotide primer pairs used in this study (table 1) were design by GeneTools software (BioTools), and their specificity was verified by blasting in the GenBank database.

**Table 1:** Primers used for the analysis of GNE and ST3Gal5 expression by Real-Time PCR.

Designation	Description	GenBank	Size (bp)	Forward primer (5′-3′)/Reverse primer (5′-3′)
*Gne*	N-acetylglucosamine 2-epimerase/N-acetylmannosamine 6-kinase	NM_015828	251	TGCCCTTCCTATGACAAACTGCTC/CGCATCACTCGAACCATCTCCT
*St3gal5*	beta-galactoside alpha-2,3-sialyltransferase 5	NM_001035228	186	GGTGTTGAGGTGGGAGGAGAG/GATGGACTAGCAGAAAGGGGTTATGAA
*Atp5o*	ATP synthase, H+ transporting mitochondrial F1 complex, O subunit	NM_138597	142	GCTATGCAACCGCCCTGTACTCTG/ACGGTGCGCTTGATGTAGGGATTC
*Hprt1*	Hypoxanthine guanine phosphoribosyl transferase 1	NM_013556	106	CAGGACTGAAAGACTTGCTCGAGAT/CAGCAGGTCAGCAAAGAACTTATAGC
*Gapdh*	Gyceraldehyde-3-phosphate dehydrogrenase	NM_008084	194	GGCTGCCCAGAACATCATCCCT/ATGCCTGCTTCACCACCTTCTTG
ADNg	Chromosome 3 genomic contig, strain C57BL/6J (HSD3B1 intron)	NT_039239	209	CACCCCTTAAGAGACCCATGTT/CCCTGCAGAGACCTTAGAAAAC

### Real-Time PCR analyses

The mRNA levels of GNE and ST3Gal5 were analyzed by the quantitative real-time polymerase chain reaction (qRT-PCR) method. RNA was isolated from fresh *Tibialis anterior* tissues (40 mg) using the RNeasy kit (Qiagen) according to the manufacturer's instructions from 7 *Gne^+/+^* and 3 *Gne^M712T/M712T^* mouse Tibialis anterior muscles. Five micrograms of total RNA were converted to cDNA by incubation with 200 U SuperScript III reverse transcriptase (Invitrogen), 50 ng random hexamers, 300 ng oligo(dT)_18_ in a reaction buffer containing 50 mM Tris-HCl, pH 8.3, 75 mM KCl, 3 mM MgCl2, 10 mM dithiothreitol (DTT), 0.5 mM dNTPs and 40 U Protector RNase inhibitor (Roche). Reaction was incubated at 25°C for 10 min, then at 50°C for 2 h and followed by treatment with 1 µg RNase A, 37°C, 30 min. PCR purification kit (Qiagen) was used to purify cDNA.

cDNA corresponding to 20 ng of the initial total RNA was used to perform fluorescence-based real-time PCR quantification using the LightCycler 480 Realtime PCR apparatus (Roche Diagnostics). The LightCycler 480 SYBR Green I Master (Roche Diagnostics) was used as described by the manufacturer. The conditions for PCR were denaturation at 95°C for 10 s, annealing at 62°C for 10 s, and elongation at 72°C for 14 s. Reading of the fluorescence signal was taken after heating at 76°C for 5 s, to avoid nonspecific signal and a melting curve was performed to assess nonspecific signal. Annealing temperature was selected based on contamination levels and melting curve results. Prior to mRNA quantification, RNA samples were verified for genomic DNA contamination.

To avoid errors due to RNA and cDNA preparation and handling, each result was corrected with three housekeeping genes, subunit O of ATPase (*Atp5o*), hypoxanthin phosphoribosyltransferase (*Hprt1*) and glyceraldehyde-3-phosphate dehydrogenase (*Gapdh*), at each assay.

Calculation of the number of copies of each mRNA was performed according to Luu-The et al. [61]: fit point and second derivative methods were used to determine a Cp that is used to calculate the number of copies of initial mRNA species in the sample using a standard.

### Western blot analysis

Proteins from *Tibialis anterior* muscles were extracted with Total Protein Extraction Kit (Chemicon) according to manufacturer's protocol. Twenty micrograms of proteins were migrated on 8% SDS-PAGE gel and transferred on nitrocellulose membrane. Primary antibody against Gne was provided by D. Darvish [47] and secondary anti-chicken-HRP was from Abcam.

### Glycosphingolipid extraction

Thirty milligrams of gastrocnemius muscles of 5 *Gne^M712T/M712T^* mice and 6 *Gne^+/+^* mice were extracted using a modification of the Svennerholm and Fredman method [62] that gave maximum recovery of gangliosides. For each of these mice, three replicates of ganglioside extraction (followed by enzymatic digestion and normal-phase HPLC analysis) were conducted per gastrocnemius muscle, in order to get the most accurate measure of ganglioside levels.

Muscle tissues were homogenized with 750 µl of Milli-Q water. The homogenate was poured in 2.7 ml of methanol, stirred and 1.35 ml of chloroform was added. After shaking, mixture was centrifuged at 2000 g for 15 min. Supernatant was kept aside whereas residue was re-extracted by addition of 2.5 ml chloroform/methanol/Milli-Q water (4/8/3), shaking and centrifugation at 2000 g for 15 min. The two extracts were combined and separated in two phases by addition of 1.3 ml Milli-Q water. After centrifugation (2000 g, 15 min), aqueous phase was removed for further processing and organic phase was re-extracted with 750 µl methanol and 500 µl Milli-Q water. After another centrifugation, both aqueous phases were pooled. Chloroform traces were eliminated under a stream of nitrogen before lyophilization of the sample.

Purification of gangliosides was obtained by solvent partition in di-isopropylether/1-butanol/Milli-Q water (60∶40∶50, v/v/v), modified and adapted from Ladisch and Gillard [63]. Basically, the samples were resuspended in two volumes (vol) of di-isopropylether/1-butanol (60/40) by vortexing and sonicating. One vol of Milli-Q water and the mixture was then stirred for several minutes. After centrifugation (2000 g, 15 min), the aqueous phase containing the gangliosides was carefully transferred in a new tube using a Pasteur pipet and a reextraction on organic phase was made by addition of 0,5 vol of Milli-Q water. Aqueous phases were combined, traces of organic solvents were evaporated under a stream of nitrogen and the sample was lyophilized. Extracted gangliosides were stored under nitrogen at –80°C in chloroform/methanol (1/1).

### Endoglycoceramidase digestion

Total glycosphingolipids from mouse muscle were dried under a stream of nitrogen and then digested with 3 mU of rEGCase II (Takara Bio Inc) in 50 µl incubation buffer (50 mM sodium acetate, pH 5.0, 0,4% TRITON-X100), for 18 h at 37°C. One unit is defined as the amount of enzyme that hydrolyzes 1 µmol of ganglioside asialo-GM1, per minute at 37°C (according to the manufacturer sheet).

### Carbohydrate fluorescent labeling and analysis by Normal Phase-HPLC (NP-HPLC)

Oligosaccharides cleaved from gangliosides were derivatized with anthranilic acid, according to Neville et al. [49]. This protocol consists in completing the sample digested by rEGCase II to 60 µl with water. 160 µl of labeling solution (anthranilic acid 30 mg/ml, sodium acetate trihydrate 4% w/v, boric acid 2% w/v and sodium cyanoborohydride 45 mg/ml, in methanol) were then added. The mix was placed at 80°C for 60 min. The reaction was allowed to cool to room temperature before addition of 1 ml acetonitrile/Milli-Q water (97/3, v/v). Purification with Discovery DPA-6S columns was conducted as follows: after pre-equilibration of the column with 2×1 ml acetonitrile, samples were loaded on the column. The column was then washed with 4×1 ml acetonitrile/Milli-Q water (99/1, v/v) and 0.5 ml acetonitrile/Milli-Q water (97/3, v/v). Labeled oligosaccharides were eluted with 2×0.6 ml Milli-Q water and, if needed, stored at –20°C. Before injections, samples were lyophilized and resuspended in Milli-Q water/acetonitrile (2/8, v/v).

NP-HPLC of purified labeled oligosaccharides was conducted using a 4.6×250 mm TSK gel amide-80 column (Tosoh Bioscience LLC), on Alliance 2695 Waters System (Milford, MA, USA) coupled with Model 474 Waters fluorescence detector set at Ex_360 nm and Em_425 nm, under control of Empower Waters software. Gradient solvents and analysis conditions were adapted from Neville et al. (2004): solvent A (20% 100 mM ammonium acetate, pH 3.85, in Milli-Q water and 80% acetonitrile) and solvent B (20% 100 mM ammonium acetate, pH 3.85, in Milli-Q water, 60% Milli-Q water, and 20% acetonitrile). Conditions of analysis were: time t = 0 min (*t*0), 86% solvent A (0.8 ml/min); *t*6, 86% solvent A (0.8 ml/min); *t*35, 54,7% solvent A (0.8 ml/min); *t*37, 5% solvent A (1 ml/min); *t*39, 5% solvent A (1 ml/min); *t*41, 86% solvent A (1 ml/min); *t*55, 86% solvent A (0.8 ml/min).

Standard gangliosides such as GM3 or mix of monosialylgangliosides (Matreya LLC) were used to identify peaks.

### Statistical tests

Fisher exact test (permutation test) was conducted to assess significant difference between GNE and ST3Gal5 mRNA expression. Student T test was used to compare muscle and kidney GM3 levels of *Gne^M712T/M712T^* and *Gne^+/+^* mouse samples.
